# Race/Ethnicity, Human Papillomavirus Vaccination Status, and Papanicolaou Test Uptake Among 27–45-Year-Old Women: A Cross-Sectional Analysis of 2019–2022 Behavioral Risk Factor Surveillance System Data

**DOI:** 10.1089/whr.2024.0170

**Published:** 2025-02-11

**Authors:** Elinita Pollard, Minjee Lee, Alice W. Lee, Mary A. Gerend, Meng-Han Tsai

**Affiliations:** ^1^ Georgia Prevention Institute, Augusta University, Augusta, Georgia, USA.; ^2^ Center for Health Equity Transformation, University of Kentucky, Lexington, Kentucky, USA.; ^3^ Department of Population Science and Policy, Southern Illinois University School of Medicine, Springfield, Illinois, USA.; ^4^ Department of Public Health, California State University, Fullerton, Fullerton, California, USA.; ^5^ Department of Behavioral Sciences and Social Medicine at the Florida State University College of Medicine, Tallahassee, Florida, USA.; ^6^ Cancer Prevention, Control, & Population Health Program, Georgia Cancer Center, Augusta University, Augusta, Georgia, USA.

**Keywords:** HPV vaccination, cervical cancer prevention, Pap test, Behavioral Risk Factor Surveillance System

## Abstract

**Purpose:**

The human papillomavirus (HPV) vaccine was recently approved for 27–45-year-olds. We examined the association between HPV vaccination status and having an up-to-date Papanicolaou (Pap) test for 27–45-year-old women across racial/ethnic groups.

**Methods:**

We conducted a cross-sectional analysis using 2019–2022 Behavioral Risk Factor Surveillance System data. We performed weighted multivariable logistic regressions to examine the association between being unvaccinated, initiating, and completing the HPV vaccine series and Pap test uptake.

**Results:**

Among 7,052 women, non-Hispanic White (NHW) women had the highest rate of HPV vaccine series completion (14.0%). Non-Hispanic Black (NHB) had the lowest rate of HPV vaccine series completion (9.2%) and the highest rate of up-to-date Pap tests (71.2%). Non-Hispanic Other (NHO) women had the lowest rate of up-to-date Pap tests (52.1%). Completing the HPV vaccine series was associated with increased odds of having an up-to-date Pap test (odds ratio [OR]: 1.66 95% confidence interval [CI]: 1.23–2.24). Among Hispanic and NHW women, HPV vaccine series completion was associated with increased odds of up-to-date Pap testing (Hispanic: OR: 2.16, 95% CI: 1.02–4.58; NHW: OR: 1.49, 95% CI: 1.01–2.21). HPV vaccine series initiation was associated with up-to-date Pap tests for NHB (OR: 2.75, 95% CI: 1.19–6.34) and NHO women (OR: 3.15, 95% CI: 1.56–6.37).

**Conclusions:**

Unvaccinated women had decreased odds of up-to-date Pap testing. Shared clinical decision-making should be utilized to help 27–45-year-old women decide if they want to receive the HPV vaccine; culturally tailored efforts should be made to improve utilization of Pap testing across racial/ethnic groups.

## Introduction 

Hispanic, non-Hispanic Black (NHB), and non-Hispanic Other (NHO) (*e.g.,* American Indian/Alaskan Native [AIAN], Native Hawaiian/Other Pacific Islander [NHOPI]) women are more likely to develop, be diagnosed at a later stage, and die from cervical cancer (CC) than non-Hispanic White (NHW) women despite the availability of effective prevention techniques such as Papanicolaou (Pap) tests and the human papilloma virus (HPV) vaccine.^1–7^ Currently, the United States Preventative Services Task Force (USPSTF) recommends 21–65-year-old women who undergo Pap testing as a form of CC screening do so once every 3 years. Women who are 30–65 have the option of being tested every 5 years with a high-risk human papillomavirus (hrHPV) test alone or in combination with a Pap test (*i.e.,* cotesting). They advise against screening for women who have had a hysterectomy.^2^ The Centers for Disease Control and Prevention’s (CDC) Advisory Committee on Immunization Practices (ACIP) recommends routine HPV vaccination for 11- and 12-year-olds. Individuals vaccinated before their 15^th^ birthday receive two doses and individuals vaccinated after their 15^th^ birthday through the catchup age of 26 should receive three doses.^8^ In 2018, the Food and Drug Administration approved the HPV vaccine series for 27–45-year-olds.^8,9^ In 2019, ACIP decided clinicians should engage in shared clinical decision-making (SCDM) with 27–45-year-olds to decide if they would like to receive the HPV vaccine as it is not recommended for everyone who is older than 26.^8^

Receiving Pap tests and completing the HPV vaccine series are highly effective strategies for reducing CC incidence rates,^10,11^ but there is racially/ethnically disparate use of each. McDaniel and colleagues found Hispanic, AIAN, Asian, and NHOPI women had 0.73, 0.66, 0.17, and 0.34 decreased odds of having an up-to-date Pap test than NHW women, respectively.^12^ Moreover, evidence has shown Hispanic and NHB women had 0.24 and 0.58 decreased odds of completing the HPV vaccine series than NHW women, respectively.^13^ Recent studies have shown women HPV vaccination is associated with being screened for CC, regardless of the screening method.^14,15^ Nationally representative survey data showed unvaccinated women had a decreased odds of having a recent Pap test than women who initiated the vaccine series; NHW women who were unvaccinated against HPV had a 0.93 decreased odds of having an up-to-date Pap test than NHW women who initiated the vaccine series, and NHO women who were unvaccinated had a 0.78 decreased odds of up-to-date Pap tests compared NHO women who initiated the series.^16^

Although the association between, Pap test uptake, and HPV vaccination has been examined previously, only one study has focused primarily on this relationship while considering race/ethnicity.^14–16^ However, the relationship between HPV vaccination “completion” (*i.e.,* three doses of the HPV vaccine series) and Pap test uptake has not been examined across different racial/ethnic groups or women older than the routinely recommended vaccination age. It is imperative to understand the differential uptake of HPV vaccination and Pap testing because it may have potential to improve HPV vaccination completion and Pap test use adherence simultaneously for early detection of CC. Thus, the primary aim of this study was to elucidate the relationship between race/ethnicity, HPV vaccine series uptake (*i.e.,* unvaccinated, initiated, completed), and Pap test uptake among 27- to 45-year-old women.

## Methods

### Study design

We utilized 2019–2022 data from the CDC’s Behavioral Risk Factor Surveillance System (BRFSS). BRFSS is a cross-sectional telephone survey administered across all 50 states, the District of Columbia, and three US territories.^17^ BRFSS uses a multistage cluster sampling technique to produce estimates representative of the US population. Data were collected using a survey questionnaire that included modules related to CC screening, HPV vaccination, demographics, tobacco use, alcohol consumption, health status, chronic health conditions, and healthcare access from adults aged ≥18 years residing in the United States.^18^ The respective health departments from each state granted Institutional Review Board (IRB) approval for the distribution and collection of data and verbal consent as directed by BRFSS. BRFSS methods, sample selection, including the weighting procedure, are described elsewhere.^19^ Because the current study uses publicly available, deidentified data, it is considered exempt from IRB review at Augusta University.

### Study participants

**FIG. 1.**
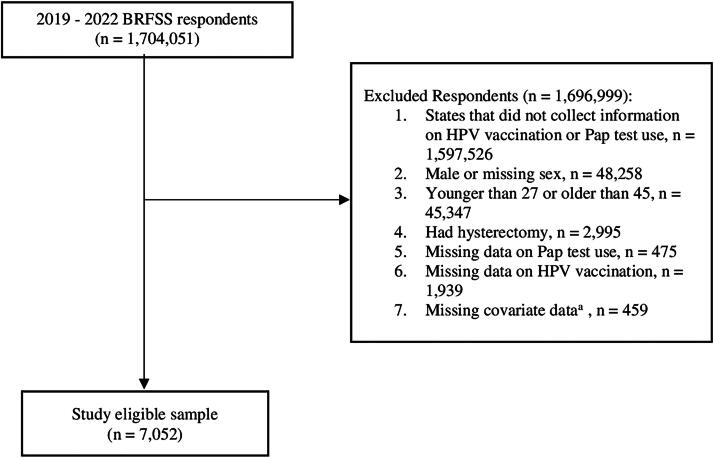
Sample Selection Flowchart. ^a^Covariates with missing data included age, education, marital status, smoker status, alcohol consumption within the last 30 days, general health, chronic disease condition(s), insurance status, routine healthcare provider, and time since last routine checkup. BRFSS, Behavioral Risk Factor Surveillance System; HPV, human papilloma virus.

In total, 1,704,051 people responded to BRFSS from 2019 to 2022. We excluded respondents in states that did not collect information on CC screening and HPV vaccination (*n* = 1,597,526; Supplementary Table S1) or reported their sex as male (*n* = 48,258). In line with ACIP and USPSTF recommendations, we excluded women who were within the routinely recommended HPV vaccination age (*i.e.,* age <27) and who were older than 45 (*n* = 45,347) and women who reported having a hysterectomy (*n* = 2,995).^2,8^ Women missing information regarding Pap test uptake (*n* = 475), HPV vaccination status (*n* = 1,939), or at least one covariate (*i.e.,* age, education, marital status, smoker status, alcohol consumption within the last 30 days, general health, chronic disease condition(s), insurance status, routine healthcare provider, and time since last routine checkup; *n* = 459) were also excluded. As a result, our final sample included 7,052 women (Fig. 1).

### Outcome of interest

Our primary outcome of interest was up-to-date Pap test uptake (yes or no) based on the USPSTF screening recommendations.^2^ Women who indicated they received their last Pap test within the past 3 years were considered to have an up-to-date Pap test, whereas women who indicated it had been three or more years ago were not considered to have an up-to-date Pap test. In 2019 and 2020, women were asked “How long has it been since your last Pap test?” In 2021 and 2022, women were asked “How long has it been since you had your last CC screening test?” followed by, “At your most recent CC screening, did you have a Pap test?” and “At your most recent CC screening, did you have an H.P.V. test?” Women who indicated their last CC screening test was within the past 3 years but not *via* Pap test were excluded as it was unclear when they had their last Pap test. Due to the inclusion of 27–29-year-olds, we did not consider other forms of CC screening (*i.e.,* hrHPV and cotesting) that are not recommended for women under 30 years of age based on USPSTF guidelines.^2^

### Exposures of interest

Our main predictor variables were race/ethnicity (Hispanic, NHB, NHO, or NHW) and HPV vaccination status (unvaccinated, initiated vaccine series, completed vaccine series). Due to small sample sizes, NHO women included those who reported their race/ethnicity as non-Hispanic AIAN, non-Hispanic Asian, or any other race/ethnicity. Two BRFSS questions were used to ascertain HPV vaccination status. Women were asked, “Have you ever had an HPV vaccination?” Women who responded with “no” were considered to have received 0 shots (termed as unvaccinated). Women who answered yes were then asked, “How many HPV shots did you receive?” BRFSS categorized responses as 1–2 or 3 shots. ACIP approved a two-dose schedule for those vaccinated before their 15^th^ birthday in 2016,^8,20^ but every woman in the current study was older than 15 at the time. Thus, every respondent would need three doses of the HPV vaccine to complete the HPV vaccine series. As such, we considered individuals who received 0 shots to be unvaccinated, those who received 1–2 shots to have initiated the HPV vaccine series, and those who received three shots to have completed the HPV vaccine series.

### Covariates

We included sociodemographic characteristics, health-related factors, healthcare access, and survey year as covariates. Sociodemographic characteristics included age (27–29, 30–34, 35–39, and 40–45 years), annual household income (less than $25,000, $25,000–$50,000, $50,000 or more, unknown), education (high school graduate or lower, some college, and college graduate), and marital status (married, divorced, and other). Those who were widowed, separated, never married, or a member of an unmarried couple were considered “other” for marital status. We considered if women reported being a current smoker (yes or no), alcohol consumption within the last 30 days (yes or no), having “good or better” or “fair or poor” health, and the number of chronic disease conditions reported (0, 1–2, 3 or more). Women were considered to have at least one chronic disease condition if they reported ever being told they had coronary heart disease or myocardial infarction, stroke, asthma, any form of cancer, chronic obstructive pulmonary disease, emphysema or chronic bronchitis, arthritis, a depressive disorder, or kidney disease. Finally, healthcare access included insurance status (yes or no), having a routine healthcare provider (yes or no), and time since last routine checkup (within the past year or longer than a year ago). We also included the survey year (2019, 2020, 2021, and 2022) as a covariate.

### Statistical analyses

We conducted weighted analyses based on the CDC’s recommendations for analysis using BRFSS data.^21^ A cross-tabulation of frequencies and weighted percentages was used to examine differences in up-to-date Pap test, race/ethnicity, HPV vaccination status, sociodemographic factors, health-related factors, and healthcare access as well as survey year. Then, we assessed differences in up-to-date Pap tests by race/ethnicity with bivariate analyses using a weighted Rao-Scott chi square test. We examined bivariate differences in sociodemographic factors, health-related factors, healthcare access, and survey year by up-to-date Pap test and race/ethnicity. Finally, multivariable logistic regressions were performed to examine the relationships among race/ethnicity, the number of HPV vaccination shots received, and Pap test use, adjusting for sociodemographic characteristics, health-related factors, healthcare access, and survey year. Subpopulation analyses within each racial/ethnic group were also conducted to examine the association between HPV vaccination doses received and Pap test use adjusting for sociodemographic characteristics, health-related factors, healthcare access, and survey year. Due to changes in BRFSS phrasing regarding Pap testing, conducted supplementary multivariable analyses stratified by year (*i.e.,* 2019–2020 and 2021–2022). All results were reported as adjusted odds ratios (ORs) and the associated 95% confidence intervals (CIs). Differences were considered significant at *p* value <0.05 using two-sided probability tests. SAS Version 9.4, SAS Institute Inc., Cary, North Carolina was used for all analyses.

## Results

### Descriptive results

Overall, most women in the current sample had a Pap test in the past 3 years (67.7%) but were unvaccinated against HPV (80.1%). There were significant racial/ethnic differences in the frequency of up-to-date Pap test uptake (*p* < 0.001) and HPV vaccination uptake (*p* < 0.01); NHB women had the highest percentage of up-to-date Pap test uptake (71.2%), followed by NHW (70.7%), Hispanic (63.1%), and NHO (52.1%) women. NHW women had the highest rate of HPV vaccine series completion (14.0%), followed by NHO (11.6%), Hispanic (9.3%), and NHB (9.2%) women (Table 1).

**Table 1.** Pap Test Uptake, HPV Vaccination, Sociodemographic Characteristics, and Health-Related Factors by Race/Ethnicity

	Total (*n* = 7,052)	Hispanic (*n* = 1,091, 18.8%)	NHB (*n* = 1,390, 22.2%)	NHO (*n* = 881, 9.0%)	NHW (*n* = 3,690, 50.0%)	*p* value
*N* (%)^a^						
Pap test						<0.001
Yes	4,848 (67.7%)	701 (63.1%)	1,016 (71.2%)	440 (52.1%)	2,691 (70.7%)	
No	2,204 (32.3%)	390 (36.9%)	374 (28.2%)	441 (47.9%)	999 (29.3%)	
HPV vaccination status						0.007
Unvaccinated	5,494 (80.1%)	862 (83.3%)	1,142 (80.9%)	634 (79.4%)	2,856 (78.7%)	
Initiated	620 (8.0%)	112 (7.4%)	105 (9.9%)	103 (9.9%)	300 (7.3%)	
Completed	938 (11.8%)	117 (9.3%)	143 (9.2%)	144 (11.6%)	534 (14.0%)	
Sociodemographic characteristics						
Age						0.477
27–29	871 (14.2%)	184 (15.8%)	160 (15.5%)	122 (17.4%)	405 (12.4%)	
30–34	1,716 (29.2%)	267 (30.4%)	343 (28.8%)	213 (27.2%)	893 (29.3%)	
35–39	1,994 (24.6%)	282 (22.3%)	385 (23.5%)	250 (26.5%)	1,077 (25.7%)	
40–45	2,471 (32.0%)	358 (31.5%)	502 (32.2%)	296 (28.9%)	1,315 (32.7%)	
Education						<0.001
≤High school	1,749 (32.3%)	501 (56.1%)	395 (31.6%)	178 (18.7%)	674 (26.2%)	
Some college	1,780 (28.8%)	242 (20.0%)	421 (34.9%)	226 (27.7%)	891 (29.5%)	
College graduate	3,523 (38.9%)	348 (24.0%)	573 (33.6%)	477 (53.5%)	2,125 (44.3%)	
Marital status						<0.001
Divorced	658 (8.0%)	96 (7.7%)	146 (29.5%)	74 (7.1%)	342 (7.8%)	
Married	3,618 (52.3%)	466 (44.3%)	396 (9.3%)	453 (63.0%)	2,303 (63.5%)	
Other^b^	2,776 (39.7%)	529 (48.0%)	848 (61.2%)	354 (29.9%)	1,045 (28.7%)	
Income						<0.001
<$25,000	1,292 (19.8%)	356 (33.0%)	396 (28.3%)	131 (12.6%)	409 (12.3%)	
$25,000–$50,000	1,368 (18.6%)	214 (18.8%)	396 (26.4%)	183 (16.8%)	575 (15.4%)	
$50,000 or more	3,567 (46.9%)	334 (26.0%)	462 (34.8%)	470 (53.8%)	2,301 (59.0%)	
Unknown^c^	825 (14.6%)	187 (22.2%)	136 (10.5%)	97 (16.8%)	405 (13.2%)	
Health-related factors						
Current smoker						<0.001
No	6,028 (84.9%)	994 (94.2%)	1,199 (86.4%)	784 (93.5%)	3,051 (79.1%)	
Yes	1,024 (15.1%)	97 (5.8%)	191 (13.6%)	97 (6.5%)	639 (20.9%)	
Alcohol consumption						<0.001
No	3,015 (45.7%)	559 (55.2%)	629 (44.1%)	447 (59.3%)	1,380 (40.3%)	
Yes	4,037 (54.3%)	532 (44.8%)	761 (55.9%)	434 (40.7%)	2,310 (59.7%)	
General health status						0.000
Fair/poor	777 (10.4%)	168 (12.6%)	206 (13.5%)	89 (6.5%)	314 (91.2%)	
Good/better	6,275 (89.6%)	923 (87.4%)	1,091 (86.5%)	792 (93.5%)	3,376 (8.8%)	
Chronic disease conditions						<0.001
0	3,827 (56.4%)	693 (71.5%)	756 (54.4%)	531 (63.2%)	1,847 (50.4%)	
1–2	2,782 (37.9%)	354 (26.1%)	542 (40.2%)	301 (33.0%)	1,585 (42.2%)	
3 or more	443 (5.6%)	44 (2.4%)	92 (5.4%)	49 (3.8%)	258 (7.3%)	
Healthcare access						
Insurance						<0.001
No	1,011 (18.4%)	404 (43.7%)	186 (14.5%)	65 (8.2%)	356 (12.5%)	
Yes	6,041 (81.6%)	687 (56.3%)	1,204 (85.5%)	816 (91.8%)	3,334 (87.5%)	
Healthcare provider						<0.001
No	1,394 (22.9%)	390 (39.4%)	218 (18.2%)	143 (21.4%)	643 (19.0%)	
Yes	5,658 (77.1%)	701 (60.6%)	1,172 (81.8%)	738 (78.6%)	3,047 (81.0%)	
Last routine checkup						<0.001
≥2 years	1,823 (27.2%)	304 (29.7%)	244 (18.2%)	240 (29.2%)	1,035 (29.9%)	
Within the past year	5,229 (72.8%)	787 (70.3%)	1,146 (81.8%)	641 (70.8%)	2,655 (70.1%)	
Response year						<0.001
2019	500 (10.8%)	83 (7.9%)	120 (15.2%)	36 (8.0%)	261 (10.5%)	
2020	3,616 (52.7%)	508 (54.5%)	772 (51.2%)	257 (43.5%)	2,079 (54.3%)	
2021	1,290 (22.3%)	229 (21.2%)	359 (26.8%)	97 (18.0%)	605 (21.5%)	
2022	1,646 (14.2%)	271 (16.4%)	139 (6.9%)	491 (30.5%)	745 (13.7%)	

^a^ Weighted Rao-Scott chi square tests were used. Data shown as frequencies and weighted percentages.

^b^ Other refers to individuals who were a member of an unmarried couple, never married, separated, or widowed.

^c^ Unknown refers to individuals who did not report their income.

HPV, human papilloma virus; NHB, non-Hispanic Black; NHO, non-Hispanic Other; NHW, non-Hispanic White; Pap, Papanicolaou.

Among Hispanic and NHW women, there was a significant difference in Pap test uptake based upon HPV vaccination status (*p* < 0.01, *p* < 0.001, respectively). Hispanic women who completed (*i.e.,* received three doses) the HPV vaccine series had the highest prevalence of Pap test uptake (81.6%), whereas those who initiated (*i.e.,* received 1 or 2 doses) the HPV vaccine series had the lowest rate Pap test uptake (60.7%). We observed the same pattern for NHW women (completed: 77.5%, initiated: 63.5%). The association between HPV vaccination status and Pap test uptake was not significant for NHB or NHO women (Table 2).

**Table 2.** HPV Vaccination, Sociodemographic Characteristics, and Health-Related Factors by Pap Test Utilization and Race/Ethnicity

	Hispanic Pap test (*n* = 701)	*p* value^b^	NHB Pap test (*n* = 1,016)	*p* value^b^	NHO Pap test (*n* = 440)	*p* value^b^	NHW Pap test (*n* = 2,691)	*p* value^b^
*N* (%)^a^								
HPV vaccination status		0.007		0.889		0.747		0.008
Unvaccinated	544 (61.2%)		824 (70.9%)		291 (51.5%)		2,076 (70.2%)	
Initiated	68 (60.7%)		75 (70.2%)		58 (58.7%)		196 (63.5%)	
Completed	89 (81.6%)		117 (74.2%)		91 (51.4%)		419 (77.5%)	
Sociodemographic characteristics								
Age		0.677		0.121		0.135		0.305
27–29	105 (58.2%)		113 (65.6%)		40 (47.3%)		291 (68.2%)	
30–34	187 (67.7%)		236 (66.8%)		99 (47.5%)		598 (68.1%)	
35–49	172 (62.6%)		277 (72.8%)		122 (46.9%)		810 (71.7%)	
40–45	237 (61.3%)		390 (76.5%)		179 (64.2%)		992 (73.2%)	
Education		0.000		0.082		0.029		<0.001
≤High school	293 (55.5%)		259 (66.4%)		62 (36.8%)		401 (57.6%)	
Some college	170 (75.0%)		321 (70.3%)		109 (47.2%)		627 (69.4%)	
College graduate	238 (70.9%)		436 (76.6%)		269 (60.0%)		1,663 (79.3%)	
Marital status		0.444		0.478		0.032		0.000
Divorced	64 (49.1%)		103 (65.1%)		43 (45.2%)		248 (71.7%)	
Married	311 (34.4%)		294 (74.1%)		231 (43.1%)		1,732 (73.6%)	
Other^b^	326 (37.3%)		619 (70.6%)		166 (58.5%)		711 (64.0%)	
Income		0.220		0.008		0.975		<0.001
<$25,000	220 (59.8%)		281 (64.6%)		53 (50.4%)		258 (58.4%)	
$25,000–$50,000	137 (55.4%)		278 (67.0%)		86 (53.1%)		381 (67.8%)	
$50,000 or more	234 (71.5%)		367 (78.9%)		250 (53.1%)		1,794 (76.2%)	
Unknown^c^	110 (64.6%)		90 (73.6%)		51 (49.4%)		258 (60.9%)	
Health-related factors								
Current smoker		0.291		0.998		0.607		<0.001
No	637 (62.7%)		876 (71.2%)		390 (52.4%)		2,286 (73.9%)	
Yes	64 (69.5%)		140 (71.1%)		50 (47.9%)		405 (58.7%)	
Alcohol consumption		0.150		0.125		0.217		0.001
No	338 (59.0%)		446 (67.8%)		207 (49.2%)		923 (65.7%)	
Yes	363 (68.0%)		570 (73.8%)		233 (56.3%)		1,768 (74.1%)	
General health status		0.994		0.222		0.882		<0.001
Fair/poor	100 (63.0%)		140 (64.9%)		44 (50.9%)		193 (55.7%)	
Good/better	601 (63.1%)		876 (72.1%)		396 (52.2%)		2,498 (72.2%)	
Chronic disease conditions		0.122		0.364		0.449		0.033
0	442 (60.5%)		555 (73.1%)		249 (50.3%)		1,376 (73.4%)	
1–2	225 (69.6%)		397 (68.1%)		162 (56.9%)		1,141 (67.4%)	
3 or more	34 (68.8%)		64 (73.6%)		29 (41.6%)		174 (71.6%)	
Healthcare access								
Insurance		0.019		0.799		0.057		<0.001
No	239 (55.7%)		126 (69.9%)		31 (40.2%)		191 (54.2%)	
Yes	462 (68.8%)		890 (71.4%)		409 (53.2%)		2,500 (73.1%)	
Healthcare provider		0.065		0.031		0.169		<0.001
No	227 (56.4%)		138 (60.9%)		59 (42.3%)		390 (56.6%)	
Yes	474 (67.4%)		878 (73.4%)		381 (54.8%)		2,301 (74.0%)	
Last routine checkup		0.001		<0.001		<0.001		<0.001
≥2 years	164 (48.7%)		139 (75.6%)		82 (31.6%)		613 (54.9%)	
Within the past year	537 (69.1%)		877 (75.6%)		358 (60.6%)		2,078 (77.5%)	
Response year		<0.001		<0.001		<0.001		<0.001
2019	68 (82.6%)		107 (84.3%)		32 (82.4%)		210 (77.9%)	
2020	438 (76.6%)		689 (88.6%)		195 (65.9%)		1,765 (82.6%)	
2021	82 (34.3%)		152 (38.9%)		42 (45.4%)		309 (47.9%)	
2022	113 (45.9%)		68 (39.7%)		171 (28.5%)		407 (54.0%)	

^a^ Weighted Rao-Scott chi square tests were used. All weighted percentages are based on row total. Data on those without up-to-date Pap tests are not shown in the table.

^b^ Other refers to individuals who were a member of an unmarried couple, never married, separated, or widowed.

^c^ Unknown refers to individuals who did not report their income.

HPV, human papilloma virus; NHB, non-Hispanic Black; NHO, non-Hispanic Other; NHW, non-Hispanic White; Pap, Papanicolaou.

### Association between race/ethnicity, HPV vaccination status, and Pap test uptake

In the fully adjusted multivariable analyses, NHO women had a 0.44–0.54 decreased odds of having an up-to-date Pap test than NHW women (OR: 0.48, 95% CI: 0.35–0.67); there were no significant differences in Pap test uptake for Hispanic and NHB women compared to NHW women. Additionally, women who completed the HPV vaccine series had 1.66 increased odds of an up-to-date Pap test compared to women who were unvaccinated (OR: 1.66, 95% CI: 1.23–2.24). When this association was examined within each racial/ethnic subgroup, Hispanic and NHW women who completed the HPV vaccine series had a 2.16 and 1.49 increased odds of having an up-to-date Pap test, respectively (Hispanic: OR: 2.16, 95% CI: 1.02–4.56; NHW: OR: 1.49, 95% CI: 1.01–2.21); NHB and NHO women who initiated the HPV vaccine series had a 2.75 and 3.15 increased odds of having an up-to-date Pap test compared to unvaccinated women in the same racial/ethnic group, respectively (NHB: OR: 2.75, 95% CI: 1.19–6.34; NHO: OR: 3.15, 95% CI: 1.56–6.37; Table 3).

**Table 3.** Association Between HPV Vaccination Status and Up-to-Date Pap Tests by Race/Ethnicity

	Total	Hispanic	NHB	NHO	NHW
OR (95% CI)^a^					
Race					
NHW	Reference	n/a	n/a	n/a	n/a
Hispanic	1.06 (0.75–1.49)	n/a	n/a	n/a	n/a
NHB	1.05 (0.81–1.35)	n/a	n/a	n/a	n/a
NHO	**0.48 (0.35–0.67)**	n/a	n/a	n/a	n/a
HPV vaccination Status					
Unvaccinated	Reference	Reference	Reference	Reference	Reference
Initiated	1.23 (0.91–1.67)	1.07 (0.59–1.96)	**2.75 (1.19–6.34)**	**3.15 (1.56–6.37)**	0.88 (0.58–1.34)
Completed	**1.66 (1.23–2.24)**	**2.16 (1.02–4.58)**	1.50 (0.65–3.47)	1.64 (0.78–3.46)	**1.49 (1.01–2.21)**

Bold text indicates the statistical significance, *p*-value <0.05.

^a^ Weighted logistic regressions were used in all models. All models are adjusted for sociodemographic factors, health-related factors, healthcare access, and survey year (data not shown).

CI, confidence interval; HPV, human papilloma virus; n/a, not applicable; NHB, non-Hispanic Black; NHO, non-Hispanic Other; NHW, non-Hispanic White; OR, odds ratio; Pap, Papanicolaou.

### Supplementary analyses

In supplementary analyses, NHO women had a decreased odds of having a Pap test compared to NHW women (2019–2020: 0.44, 95% CI: 0.26–0.73; 2021–2022: OR: 0.54, 95% CI: 0.38–0.77). HPV vaccine series initiation was only associated with Pap test uptake from 2021–2022 (OR: 1.49, 95% CI: 1.02–2.18), but vaccine series completion was associated with an increased odds of Pap testing for both time periods (2019–2020: OR: 1.80, 95% CI: 1.05–3.09; 2021–2022: OR: 1.92, 95% CI: 1.32–2.80; Supplementary Table S2).

From 2019 to 2020, HPV vaccine series completion was marginally associated with having an up-to-date Pap test for NHO women (OR: 6.11, 95% CI: 1.00–37.25). From 2021 to 2022, HPV vaccine series initiation was associated with increased odds of having an up-to-date Pap test for NHB and NHO women (NHB: OR: 3.38, 95% CI: 1.34–8.55; NHO: OR: 3.23, 95% CI: 3.23, 95% CI: 1.37–7.59). HPV vaccine series completion was associated with increased odds of having an up-to-date Pap test for NHW women (OR: 1.86, 95% CI: 1.10–3.13; Supplementary Table S3).

## Discussion

Over half of the women in the current study had up-to-date Pap test uptake (67.7%), but we observed low rates of HPV vaccine series initiation (8.0%) and completion (11.8%). The same pattern has been observed in previous studies. Silver and Kobrin found that 27.3% of 21–36-year-old women reported being vaccinated against HPV, whereas 84.2% reported ever having a Pap test.^15^ Similarly, Sauer and colleagues reported 82.6% of women had an up-to-date Pap test, but only 15.3% of 21-to-30-year-old women were vaccinated against HPV.^16^ Lower rates of Pap testing and HPV vaccination rates in our study may have been due to differences in methodology and data sources. While we considered up-to-date Pap testing, Silver and Kobrin considered if women *ever* had a Pap test.^15^ Though BRFSS is administered in the entire United States, few states asked about both HPV vaccination and Pap testing from 2019 to 2022. However, Sauer and colleagues used data representative of the entire United States.^16^ Moreover, age is inherently a barrier to HPV vaccine uptake in our sample as HPV vaccine uptake is not recommended for every 27–45-year old.^8^

In line with previous evidence that suggested completion (*i.e.,* receiving three doses) of the HPV vaccine series is associated with having an up-to-date Pap test,^16,22^ we found women who completed the HPV vaccine series had up to a 1.92 increased odds of having an up-to-date Pap test compared to unvaccinated women. The relationship between HPV vaccination status and having an up-to-date Pap test may be due to shared factors between the two such as health literacy and provider recommendation. Health literacy is positively associated with the likelihood HPV vaccine series initiation and completion and a 1.64 increased odds of Pap test uptake^23–25^; provider recommendation has been associated with 10.1 higher odds of HPV vaccine series initiation, 5.2 higher odds of HPV vaccine series completion, and up to 2.69 higher odds of Pap test uptake.^26,27^ Evidence has shown that women who received the HPV vaccine had a higher prevalence of Pap test recommendations,^28^ presumably facilitating the relationship between HPV vaccination status and Pap test uptake.

Our findings regarding the relationship between HPV vaccination and having an up-to-date Pap test were only partially supported by previous research when race/ethnicity was considered. To our knowledge, Sauer et al. is the only other study that has primarily focused on the relationship between HPV vaccine initiation while considering racial/ethnic differences using a nationally administered survey. Their findings showed NHO and NHW women who did not initiate the HPV vaccine series had a 0.93 and 0.78 decreased odds of having an up-to-date for Pap test compared to women in the same racial/ethnic group, respectively.^16^ Similarly, *initiating* the HPV vaccine series was associated with increased odds of having an up-to-date Pap test by 3.15 times for NHO women, while completing the HPV vaccine series was associated with 1.49 greater odds of having an up-to-date Pap test for NHW women in the current study. We also found Hispanic women who completed the HPV vaccine series had an increased odds of having an up-to-date Pap test and NHB women who initiated the HPV vaccine series had a 2.75 increased odds of having an up-to-date Pap test. In contrast, Sauer and colleagues did not find a significant relationship between HPV vaccine series initiation and up-to-date Pap testing for Hispanic and NHB women.^16^ Discrepancies in our results may be due to differences in study design. Sauer and colleagues examined this association among 21–30-year-olds while we focused on women who were 27–45, an age group that was recently approved for HPV vaccination uptake in 2018.^9^ Furthermore, Sauer and colleagues did not consider the impact of HPV vaccine series *completion* across diverse racial/ethnic groups.^16^ Thus, our analysis provides an updated, more nuanced approach to understanding the relationship between HPV vaccination series status and Pap testing.

HPV uptake is associated with facilitators of Pap test uptake (*e.g.,* provider recommendation, higher levels of health literacy), regardless of race/ethnicity.^23–25,29–31^ However, racially/ethnically minoritized women disproportionately experience barriers to HPV vaccine uptake (*e.g.,* medical mistrust, lower HPV vaccine awareness) due to historical injustices and systemic racism,^29,32–40^ which is illustrated in the differential rates of HPV vaccine series uptake in our sample. Nearly twice as many NHW women completed the HPV vaccine series as NHW women who initiated the vaccine series, but within other racial/ethnic groups, there were similar rates of HPV vaccine series initiation and completion. Hirth and colleagues concluded physicians may prioritize vaccine series initiation over completion and fail to follow-up with their patients.^41^ Completing the series is more effective than initiating it.^42,43^ However, NHB and NHO women are more likely to experience barriers within the healthcare system such as losing insurance coverage and poorer quality of patient–provider communication compared to their NHW counterparts.^44,45^ Additionally, the self-reported nature of BRFSS may have led to vaccine initiation and completion rates being over or underestimated.^46^ Factors such as these may explain why initiation, rather than completion, was associated with Pap test uptake for NHB and NHO women.

Finally, we found that NHO women had decreased odds of having an up-to-date Pap test than NHW women. Results from a previous study suggest this disparity only exists among women who have not been vaccinated against HPV; compared to NHW women, NHO women did not have significantly different rates of up-to-date Pap tests among women who were vaccinated against HPV.^16^ NHO (*e.g.,* Asian, AIAN) women face unique barriers to Pap test uptake specifically such as beliefs surrounding modesty and hesitancy surrounding Western medicine, contextualized by historical injustices.^6,38,47^ Additionally, AIAN Asian American and Pacific Islander women experience systemic barriers that contribute to *both* lack of HPV vaccine and Pap test uptake compared to their NHW counterparts, including less access to healthcare and lower levels of health literacy due to inaccessible health communication.^23–25,29–31,48^

### Implications

Our findings suggest women who have not undergone primary prevention for CC *via* HPV vaccine series uptake have decreased odds of undergoing secondary CC prevention *via* Pap testing, putting them at an increased risk for adverse CC outcomes.^10,11^ Lack of provider recommendation, concerns about safety, and perceiving the vaccine as unnecessary serve as additional barriers to HPV vaccine uptake among adults.^49^ Of note, it has been estimated that initiating the vaccine series at the ages of 25, 35, and 45 reduce the vaccine’s lifetime effectiveness for CC prevention by 60%, 70%, and 90%, respectively. However, it can still decrease lifetime risk for CC up to 29% in these age groups.^50^ Thus, clinicians should engage in SCDM with patients to determine if they would like to be vaccinated in line with ACIP’s recommendations.^8^ However, emphasizing the importance of Pap testing among this age group is imperative as it may have a larger net benefit regarding CC outcomes.

Racial/ethnic disparities in utilization of CC prevention methods indicate efforts to improve HPV vaccination rates and Pap testing should aim to be mindful of racially/ethnically diverse groups’ experiences, perceptions, and beliefs among healthcare providers and researchers alike. SCDM has the potential to increase HPV vaccine initiation and completion as well as Pap test uptake among Hispanic, NHB, and NHO women specifically as it has been linked to more trust in clinicians and feelings of empowerment,^51^ and medical mistrust is a common barrier to CC prevention among these racial/ethnic groups.^36–39^ However, more research focusing on SCDM’s efficacy in increasing HPV vaccine and Pap test uptake overall and across diverse racial/ethnic groups is warranted. Rates of Pap test uptake in AIAN, Asian, and NHOPI women could potentially be improved by community-based group education, culturally sensitive education, and patient navigation.^52–54^

### Strengths and limitations

The main strength of this study is that we considered HPV vaccine series initiation and completion based on ACIP recommendations.^8^ We also examined the association between HPV vaccine series and Pap test uptake among 27–45-year-olds across different racial/ethnic groups. Our findings indicated HPV vaccination series completion is positively associated with Pap test use for CC prevention for Hispanic and NHW women who are older than the age that HPV vaccine uptake is routinely recommended. Findings from our study provide an updated, more granular analysis of this association based on contemporary recommendations and may guide primary care initiatives.

The current study is also marked by limitations. Due to the cross-sectional nature of BRFSS, temporal relationships between HPV vaccination and Pap test use across different racial/ethnic groups cannot be confirmed. Furthermore, we combined non-Hispanic AIAN and non-Hispanic Asian women with the original NHO category in BRFSS due to small sample sizes. Because the NHO group is comprised of multiple racial/ethnic groups, insights about these groups cannot be ascertained. Moreover, racial/ethnic categories such as “Asian” (*e.g.,* Korean, Japanese women) and “Hispanic” (*e.g.,* Mexican, Puerto Rican women) homogenize a variety of communities. Due to small sample sizes and data limitations (Supplementary Table S4), information regarding these specific communities could not be ascertained. Therefore, efforts should be made to understand this association among specific racial/ethnic communities. Due to BRFSS’ use of self-reported data, recall bias and social desirability may have influenced outcomes; participants may have misreported their HPV vaccination and/or Pap test behaviors.^46^ Changes in phrasing may have impacted the results; this is reflected in our results for 2019–2020 BRFSS (Supplementary Table S3). Significant results were only observed in BRFSS 2021–2022 and the entire study period. Thus, future researchers should be cognizant of this when utilizing this Pap test question across different years of data. Moreover, we did not consider if women received hrHPV testing as some participants were not eligible for it and few women receive the hrHPV testing as a form of CC screening alone.^55^ Future researchers may be interested in considering both forms of testing. Finally, BRFSS does not ask respondents at what age they received the first dose of the HPV vaccine. Future researchers may want to consider this as age-specific factors (*e.g.,* parental approval) may influence this association.

## Conclusions

Results indicate 27–45-year-old women who completed the HPV vaccine series had increased odds of having an up-to-date Pap test overall. Hispanic and NHW women who completed the HPV vaccine series as well as NHB and NHO women who initiated the HPV vaccine series also had an increased odds of having an up-to-date Pap test. Effective implementation of SCDM and use of culturally sensitive interventions may improve HPV vaccination completion and Pap test use across racially/ethnically diverse groups.

## Data Availability

The datasets generated during the current study are available in the Center for Disease Control, and Prevention repository, https://www.cdc.gov/brfss/.

## Abbreviations Used

ACIP: Advisory Committee on Immunization Practices
BRFSS: Behavioral Risk Factor Surveillance System
CC: cervical cancer
CDC: Centers for Disease Control and Prevention
HPV: Human papillomavirus
IRB: Institutional Review Board
NHO: Non-Hispanic Other
NHB: Non-Hispanic Blac
NHW: non-Hispanic White
OR: Odds ratio
Pap: Papanicolaou
SCDM: shared clinical decision-making
