# Supplementary material for: Race/Ethnicity, Human Papillomavirus Vaccination Status, and Papanicolaou Test Uptake Among 27–45-Year-Old Women: A Cross-Sectional Analysis of 2019–2022 Behavioral Risk Factor Surveillance System Data
Source: Womens Health Rep (New Rochelle). 2025 Feb 11;6(1):178–89. doi: 10.1089/whr.2024.0170 (PMC11931109; doi:10.1089/whr.2024.0170)
Supplement: Supplementary Table S1 [file whr.2024.0170_supp_table_s1.docx]

**Supplementary Table 1. Study eligible states in BRFSS 2019-2022**

| **Year** | **States that asked about HPV vaccination and Pap testing** |
| --- | --- |
| 2019 | Georgia |
| 2020 | Arkansas, Connecticut, Georgia, Illinois, Mississippi, New Jersey, North Dakota, South Carolina |
| 2021 | Georgia, Mississippi, New Jersey |
| 2022 | Arkansas, Delaware, Hawaii, New Jersey |

Abbreviations: BRFSS, Behavioral Risk Factor Surveillance System; HPV, human papillomavirus.
