# Supplementary material for: Race/Ethnicity, Human Papillomavirus Vaccination Status, and Papanicolaou Test Uptake Among 27–45-Year-Old Women: A Cross-Sectional Analysis of 2019–2022 Behavioral Risk Factor Surveillance System Data
Source: Womens Health Rep (New Rochelle). 2025 Feb 11;6(1):178–89. doi: 10.1089/whr.2024.0170 (PMC11931109; doi:10.1089/whr.2024.0170)
Supplement: Supplementary Table S2 [file whr.2024.0170_supp_table_s2.docx]

**Supplementary Table 2. Association between Race, HPV vaccination status, and up-to-date Pap tests by survey year**

|  | **2019-2020** | **2021-2022** |
| --- | --- | --- |
| **OR(95% CI)^a^** | | |
| **Race** |  |  |
| NHW | Reference | Reference |
| Hispanic | 1.27(0.71-2.30) | 0.91(0.64-1.30) |
| NHB | 1.48(0.95-2.29) | 0.74(0.53-1.03) |
| NHO | **0.44(0.26-0.73)** | **0.54(0.38-0.77)** |
| **HPV Vaccination Status** |  |  |
| Unvaccinated | Reference | Reference |
| Initiated | 1.26(0.73-2.17) | **1.49(1.02-2.18)** |
| Completed | **1.80(1.05-3.09)** | **1.92(1.32-2.80)** |

Abbreviations: NHB, non-Hispanic Black; NHO, non-Hispanic Other; NHW, non-Hispanic White; OR, odd ratio; CI, confidence interval.

^a^ Weighted logistic regressions were used in all models. All models are adjusted for sociodemographic factors, health-related factors, healthcare access, and survey year (data not shown).

^b^ Other refers to individuals who were a member of an unmarried couple, never married, separated, or widowed; Unknown refers to individuals who did not report their income.
