# Supplementary material for: Race/Ethnicity, Human Papillomavirus Vaccination Status, and Papanicolaou Test Uptake Among 27–45-Year-Old Women: A Cross-Sectional Analysis of 2019–2022 Behavioral Risk Factor Surveillance System Data
Source: Womens Health Rep (New Rochelle). 2025 Feb 11;6(1):178–89. doi: 10.1089/whr.2024.0170 (PMC11931109; doi:10.1089/whr.2024.0170)
Supplement: Supplementary Table S3 [file whr.2024.0170_supp_table_s3.docx]

**Supplementary Table 3. Association between HPV vaccination status and Pap tests by race and survey year**

|  | **2019-2020** | | | | **2021-2022** | | | |
| --- | --- | --- | --- | --- | --- | --- | --- | --- |
|  | **Hispanic** | **NHB** | **NHO** | **NHW** | **Hispanic** | **NHB** | **NHO** | **NHW** |
| **OR(95% CI)^a^** | | | | | | | | |
| **HPV Vaccination Status** |  |  |  |  |  |  |  |  |
| Unvaccinated | Reference | Reference | Reference | Reference | Reference | Reference | Reference | Reference |
| Initiated | 5.37(0.86-33.80) | 3.96(0.51-30.91) | 2.16(0.39-11.96) | 0.55(0.30-1.04) | 0.67(0.28-1.60) | **3.38(1.34-8.55)** | **3.23(1.37-7.59)** | 1.33(0.76-2.34) |
| Completed | 1.97(0.46-8.44) | 1.07(0.27-4.23) | **6.11(1.00-37.25)** | 1.76(0.92-3.38) | 2.17(0.90-5.26) | 2.40(0.98-5.88) | 2.24(090-5.56) | **1.86(1.10-3.13)** |

Abbreviations: NHB, non-Hispanic Black; NHO, non-Hispanic Other; NHW, non-Hispanic White

^a^ Weighted logistic regressions were used in all models. All models are adjusted for sociodemographic factors, health-related factors, healthcare access, and survey year (data not shown).

^b^ Other refers to individuals who were a member of an unmarried couple, never married, separated, or widowed; Unknown refers to individuals who did not report their income.
