# Supplementary material for: Race/Ethnicity, Human Papillomavirus Vaccination Status, and Papanicolaou Test Uptake Among 27–45-Year-Old Women: A Cross-Sectional Analysis of 2019–2022 Behavioral Risk Factor Surveillance System Data
Source: Womens Health Rep (New Rochelle). 2025 Feb 11;6(1):178–89. doi: 10.1089/whr.2024.0170 (PMC11931109; doi:10.1089/whr.2024.0170)
Supplement: Supplementary Table S4 [file whr.2024.0170_supp_table_s4.docx]

**Supplementary Table 4. Racial/ethnic group distribution by state**

|  | **Hispanic**  **(n = 1,091, 15.5%)** | **NHAIAN**  **(n = 51, 0.72%)** | **NHA**  **(n = 394, 5.6%)** | **NHB**  **(n = 1,390, 19.7%)** | **NHO**  **(n = 436, 6.2%)** | **NHW**  **(n = 3,690, 52.3%)** |
| --- | --- | --- | --- | --- | --- | --- |
| **N(%)** | | | | | | |
| **State** |  |  |  |  |  |  |
| Arkansas | 33(3.0%) | 5(9.8%) | 5(1.3%) | 67(4.8%) | 25(5.7%) | 380(10.3%) |
| Connecticut | 145(13.3%) | 3(5.9%) | 15(3.8%) | 69(5.0%) | 28(6.4%) | 415(11.3%) |
| Delaware | 48(4.4%) | 2(3.9%) | 3(0.8%) | 39(2.8%) | 9(2.1%) | 131(3.6%) |
| Georgia | 194(17.8%) | 9(17.7%) | 37(9.4%) | 426(30.7%) | 57(13.1%) | 769(20.8%) |
| Hawaii | 73(6.7%) | 2(3.9%) | 169(42.9%) | 11(0.8%) | 233(53.4%) | 145(3.9%) |
| Illinois | 38(3.5%) | 0(0%) | 17(4.3%) | 33(0.8%) | 1(0.2%) | 158(4.3%) |
| Mississippi | 12(1.1%) | 3(5.9%) | 4(1.0%) | 429(30.9%) | 7(1.6%) | 389(10.5%) |
| New Jersey | 536(49.1%) | 5(9.8%) | 140(35.5%) | 248(17.8%) | 67(15.4%) | 961(26.0%) |
| North Dakota | 49(0.4%) | 20(39.2%) | 2(0.5%) | 4(0.3%) | 4(0.9%) | 212(5.8%) |
| South Carolina | 8(0.7%) | 2(3.9%) | 2(0.5%) | 64(4.6%) | 5(1.2%) | 130(3.5%) |

Abbreviations: NHAIAN, non-Hispanic American Indian and Alaksa Native; NHA, non-Hispanic Asian; NHB, non-Hispanic Black; NHO, non-Hispanic Other; NHW, non-Hispanic White
